# The association between medical education accreditation and the examination performance of internationally educated physicians seeking certification in the United States

**DOI:** 10.1007/s40037-015-0183-y

**Published:** 2015-05-07

**Authors:** Marta van Zanten

**Affiliations:** Foundation for Advancement of International Medical Education and Research (FAIMER), 3624 Market Street, 19104 Philadelphia, PA USA

**Keywords:** International medical graduates, Accreditation, Caribbean

## Abstract

The purpose of this study was to investigate the performance of graduates of international medical schools who seek Educational Commission for Foreign Medical Graduates certification based on accreditation of their medical education programmes. For the self-selected population who took United States Medical Licensing Examinations during the study period (2006–2010), accreditation was associated with higher first-attempt pass rates on some examinations, especially for international medical graduates from schools located in the Caribbean region. In addition, certain essential accreditation standards were associated with better performance on all examinations. This study lends support to the value of medical education accreditation.

## Introduction

While the medical school accreditation process in the United States assures a uniform standard of quality at US medical education programmes, approximately one quarter of physicians in training and in practice in the United States graduated from medical schools located outside of the United States or Canada [1]. These graduates of international medical schools are more likely than domestically educated doctors to practice primary care and treat underserved and minority populations [2]. An increasing proportion of international medical graduates who seek to enter postgraduate training programmes and subsequent licensure in the United States graduated from medical schools located in the Caribbean. The quality of medical education at some of these schools has been questioned [3].

Accreditation systems are frequently viewed as a way to ensure the quality of medical education, although currently there is limited data linking an educational oversight mechanism to better performance of the graduates [4–6]. In addition, accreditation systems vary in the methodology, standards, and procedures used to evaluate educational programmes [7−9]. The purpose of the first phase of this study was to examine medical education accreditation practices around the world, with special focus on the Caribbean due to the increasing proportion of graduates from the region seeking to enter the US health care system, to determine the association of accreditation of medical schools with student/graduate performance on examinations. The aim of the second phase of this research was to evaluate the quality of a select group of accrediting agencies and the association of quality with student/graduate outcomes.

## Methods

All international medical graduates seeking to enter graduate training positions in the United States must first be certified by the Educational Commission for Foreign Medical Graduates. In addition to other requirements, Educational Commission for Foreign Medical Graduates certification includes passing scores on the United States Medical Licensing Examination Step 1 Basic Science, Step 2 Clinical Knowledge, and Step 2 Clinical Skills.

In the first phase, all international medical graduates taking one or more examinations leading to Educational Commission for Foreign Medical Graduates certification during the five-year study period (January 1 2006 through December 31 2010) and who graduated from, or attended at the time of testing, a school located in a country that met the accreditation inclusion criteria, were included in the study population. First-attempt pass rates for each examination were calculated based on personal variables (gender, years elapsed since graduation at the time the individual took an examination [< 3 years versus ≥ 3 years], native language [English versus all others]), and on accreditation status of an individual’s medical school. Next, separately for each examination, a generalized estimating equations model [10] was used to investigate the effect of accreditation after controlling for the personal variables. Following the assessment of accreditation on test performance on the entire global group, the global group was divided into students/graduates from medical schools located in the Caribbean (the area of focus due to the increasing proportion of international medical graduates seeking Educational Commission for Foreign Medical Graduates certification from the region), and for comparison, students/graduates from medical school not located in the Caribbean. The same analyses conducted on the global group were next conducted separately on the data from the Caribbean group and on the data from the non-Caribbean group.

In the second phase, the quality of a select group of accrediting agencies was evaluated according to the criteria determined by a panel of experts to be the most salient features of an accreditation system. Accreditation systems that used 80 % or more of the criteria were given a quality grade of A, and systems using less than 80 % of the criteria were given a grade of B.

The association between the quality of an accreditation system and student performance, as measured by first-attempt pass rates on United States Medical Licensing Examinations, was investigated in this second phase.

The Temple University Office for Human Subject Protections Institutional Review Board determined by expedited review that this study qualified for exemption status.

## Results

As of January 2011, there were 173 countries with medical schools listed in the International Medical Education Directory, of which 118 met the inclusion criteria. Of these 118 countries (global group), 77 countries had a system of accreditation and 41 countries did not have accreditation. There were 19 countries in the Caribbean group, 11 with accreditation and eight without accreditation, and 99 countries in the non-Caribbean group, 66 with accreditation and 33 without accreditation. During the study period approximately 67,000 students/graduates took Step 1 Basic Science for the first time, 55,600 took Step 2 Clinical Knowledge, and 58,200 took Step 2 Clinical Skills. Over one quarter of the test takers graduated from, or were students at, schools located in the Caribbean. For each of the three groups (global, Caribbean and non-Caribbean), Table 1 displays summary results of the association between the existence of a system of accreditation and the United States Medical Licensing Examination first-attempt pass rates of international medical graduates seeking Educational Commission for Foreign Medical Graduates certification. For the global population, better performance on Step 1 Basic Science was associated with the male gender, testing within three years of graduation, non-native English-speaking status, and attending a school located in a country with a system of accreditation. For the Caribbean population on Step 1 Basic Science, results were similar, except native English speakers outperformed non-native English speakers. After controlling for covariates, the odds of passing Step 1 Basic Science for those from accredited schools were 1.8 times greater for the global group and 4.9 times greater for the Caribbean group as compared with the odds of passing the examination on the first attempt for individuals from non-accredited schools. In contrast, in the non-Caribbean group accreditation was not associated with examination performance.

**Table 1:** Summary of the association between accreditation of medical schools and United States Medical Licensing Examination first-attempt pass rates

		Examination		
		Step 1 Basic science	Step 2 Clinical knowledge	Step 2 Clinical skills
Global (*n* countries = 118)	First-attempt pass rates for students/graduates from accredited medical schools (*n* countries = 77)	75.4 % (*n* = 56,094)	82.5 % (*n* = 47,701)	76.4 % (*n* = 49,898)
	First-attempt pass rates for student/graduate from non-accredited medical schools (*n* countries = 41)	63.3 % (*n* = 10,974)	79.6 % (*n* = 7901)	70.1 % (*n* = 8370)
	Unadjusted OR	1.8	1.2	1.4
	Adjusted OR^a^	1.8	1.3	1.3
Caribbean (*n* countries = 19)	First-attempt pass rates for students/graduates from accredited medical schools (*n* countries = 11)	74.0 % (*n* = 14,676)	76.8 % (*n* = 11,758)	85.2 % (*n* = 12,431)
	First-attempt pass rates for students/graduates from non-accredited medical schools (*n* countries = 8)	38.4 % (*n* = 3654)	59.0 % (*n* = 1510)	71.9 % (*n* = 1794)
	Unadjusted OR	4.6	2.3	2.3
	Adjusted OR^a^	4.9	2.3	2.4
Non-Caribbean (*n* countries = 99)	First-attempt pass rates for students/graduates from accredited medical schools (*n* countries = 66)	75.9 % (*n* = 41,418)	84.3 % (*n* = 35,943)	73.5 % (*n* = 37,467)
	First-attempt pass rates for students/graduates from non-accredited medical schools (*n* countries = 33)	75.8 % (*n* = 7320)	84.5 % (*n* = 6391)	69.6 % (*n* = 6576)
	Unadjusted OR	1.0	1.0	1.2
	Adjusted OR^a^	1.0	1.0	1.1

^a^After controlling for gender, years since graduation, and native language, increase in odds of a student/graduate from an accredited school passing the examination on the first attempt as compared with the odds of a student/graduate from a non-accredited school passing the examination on the first attempt. *OR* odds ratio.

Increased performance on Step 2 Clinical Knowledge for the global group was associated with the female gender, testing within three years of graduation, non-native English-speaking status, and attending a school located in a country with a system of accreditation. For the Caribbean population on Step 2 Clinical Knowledge, females, those testing closer to graduation, and native English speakers outperformed their counterparts. After controlling for covariates, the odds of passing Step 2 Clinical Knowledge for those from accredited schools were 1.3 times greater for the global group and 2.3 times greater for the Caribbean group as compared with individuals from non-accredited schools. Accreditation was not associated with examination performance for the non-Caribbean group.

For all three groups (global, Caribbean, and non-Caribbean), better performance on Step 2 Clinical Skills was associated with the female gender, testing within three years of graduation, native English-speaking status, and attending a school located in a country with a system of accreditation. After controlling for covariates, the odds of passing Step 2 Clinical Skills for those from accredited schools were 1.3 times greater for the global group, 2.4 times greater for the Caribbean group, and 1.1 times greater for the non-Caribbean group compared with individuals from non-accredited schools.

In phase two, the expert panel unanimously agreed on 14 essential standards that should be required by accrediting agencies to ensure the quality of physicians [11]. Of the accreditation systems in 18 countries that were analyzed for inclusion of the criteria, four systems, used in 10 countries, were given a grade of A (included 80 % or more of the essential standards), and eight systems, used in eight countries, were given a grade of B (included less than 80 % of the essential standards). The international medical graduates attending medical schools accredited by a system that received a grade of A performed better on Step 1 and Step 2 Clinical Skills as compared with international medical graduates attending medical schools that are accredited by a system receiving a grade B. For Step 2 Clinical Knowledge, the results were reversed. Certain essential standards were associated with better performance for all three examinations.

## Discussion

The purpose of this study was to investigate the United States Medical Licensing Examination performance of graduates of international medical schools who voluntarily seek Educational Commission for Foreign Medical Graduates certification based on variables related to the accreditation of their medical education programmes. In this study, for the self-selected population who took examinations during the study period, accreditation was associated with better performance in specific regions and for some examinations.

Of the three examinations, the existence of a system of accreditation had the strongest association with Step 1 Basic Science performance for the global and Caribbean groups. Many accreditation criteria are directly related to aspects of the preclinical phase of education. The association between accreditation and Step 2 Clinical Skills was positive for all three groups of students/graduates, although systems of accreditation may have less direct impact on student performance on clinical examinations as students’ experiences in the clinical phase are likely more varied [12]. Of the three groups, the existence of accreditation systems had the greatest association with examination performance in the Caribbean, an important finding considering the large numbers of international medical graduates educated in this region seeking Educational Commission for Foreign Medical Graduates certification and ultimately treating US patients [13].

The quality of accrediting agencies, as determined by the number of essential elements utilized in the systems, was positively associated with performance for Step 1 Basic Science and Step 2 Clinical Skills, but not Step 2 Clinical Knowledge. The finding supporting the importance of a high-quality accreditation system on Step 2 Clinical Skills performance is important due to the purpose of this examination in evaluating a physician’s skills in a real-world setting.

This study lends some support to the value of accreditation [14]. Due to the substantial resources needed to design and implement accreditation processes, these results provide some positive evidence beyond face validity, especially in the Caribbean region, that quality assurance oversight of educational programmes is associated with the production of more highly skilled physicians, which in turn should improve the health care of patients in the United States and around the world.

Tips for PhD students

- Make friends with your programme’s secretarial and administrative support staff. These individuals will be very useful to you in helping navigate requirements unrelated to your actual research, especially the inevitable institutional red tape.
- Your required and elective course work may cover diverse fields, but try to focus your course assignments on topics that will later be relevant to your dissertation. Even at the beginning of your doctoral programme when you may not yet have an actual dissertation idea, attempt to focus your course work on one similar topic area or theme of inquiry.
- Familiarize yourself with the university’s Graduate School policies for doctoral students. The Graduate School’s rules will usually supersede an individual programme’s procedures, especially for matters related to the university’s acceptance of your thesis and your eligibility to officially graduate.
- Recognize that reaching the stage of defending your dissertation does not necessarily indicate that you have finished. Even if your committee determines that you have passed your defense, they may request additional analyses. Be prepared to quickly make the requested changes within the short timeframe allowed for revisions.
- When purchasing academic regalia, splurge and rent or buy an actual velvet gown. The inexpensive gowns for sale through the university bookstore are made of recycled soda bottles and are known to rip at inopportune moments and disintegrate if worn in the rain. Also, if allowed, purchase a round hat or ‘tam’, as opposed to a square academic hat, or ‘mortar board’. Wearing a mortar board will make it difficult for your Chair to place the academic hood over your head during the graduation ceremony, especially if your Chair is shorter than you.
